# Evaluation of cardiac autonomic control during the 6-min walk test in women with systemic sclerosis

**DOI:** 10.1186/s13104-023-06522-9

**Published:** 2023-09-30

**Authors:** Nathália Alves de Oliveira Saraiva, Iasmim de Oliveira Farias, Brenda Mesquita dos Santos, Rosemere Saldanha Xavier, Agnaldo José Lopes

**Affiliations:** 1 Post-Graduation Programme in Rehabilitation Sciences, Centro Universitário Augusto Motta (UNISUAM), Rua Dona Isabel, 94, Bonsucesso, Rio de Janeiro, 21032-060 Brazil; 2 Faculty of Physiotherapy, Centro Universitário Augusto Motta (UNISUAM), Av. Paris, 84, Bonsucesso, Rio de Janeiro, 21041-020 Brazil; 3 Post-Graduation Programme in Local Development, Centro Universitário Augusto Motta (UNISUAM), Rua Dona Isabel, 94, Bonsucesso, Rio de Janeiro, 21032-060 Brazil

**Keywords:** Systemic sclerosis, Exercise, Autonomic nervous system, Heart rate

## Abstract

**Objective:**

To evaluate the association between sympathovagal balance and exercise performance, as measured by the 6-min walk test (6MWT), in women with systemic sclerosis (SSc) without cardiac involvement.

**Results:**

This was a cross-sectional study in which 69 women with SSc [median age 51 (40–63 years)] without cardiac involvement underwent the 6MWT. Throughout the 6MWT, heart rate variability (HRV) was assessed using dedicated software.

**Methods:**

The median 6-min walking distance (6MWD) was 451 (392–498) meters, and 29 (42%) participants did not achieve 80% of the predicted value for healthy adults. Desaturation during the 6MWT (SpO_2_ ≤ 4%) was observed in 10.1% of participants. Significant correlations were observed between the 6MWD and the following HRV parameters: number of successive normal-to-normal RR interval differences > 50 ms (*r*_*s*_=-0.397, *P* = 0.013), low-frequency range (*r*_*s*_=0.374, *P* = 0.023), high-frequency range (*r*_*s*_=-0.372, *P* = 0.023), and parasympathetic nervous system index (*r*_*s*_=-0.342, *P* = 0.045).

**Conclusion:**

In women with SSc, there is an interrelationship of the 6MWD with both vagal withdrawal and sympathetic hyperactivation. This relationship between autonomic imbalance and worse exercise performance might increase cardiovascular risk, even in patients without apparent cardiac involvement. Control of the heart by the autonomic nervous system may be a potential target for treating patients with SSc.

## Introduction

Systemic sclerosis (SSc) is a complex immune-mediated disease of the connective tissue characterized by progressive fibrosis due to collagen deposition [1]. Although SSc is a multisystemic condition, cardiopulmonary manifestations are responsible for up to 85% of mortality [2]. In the heart, all structures can be affected by SSc, leading to inflammation, oxidative stress, vascular damage, and fibrosis [3]. The main underlying mechanism seems to be impairment of the microcirculation, with abnormal vasoreactivity due to dysfunction of the autonomic nervous system (ANS) [4]. In fact, ANS dysfunction in patients with SSc is associated with the risk of arrhythmia and mortality, and it is an early marker of SSc progression that may help to identify subclinical cardiac involvement and precede cardiac fibrosis [5]. In SSc, it is believed that vagal withdrawal together with sympathetic hypertonia is a compensatory mechanism secondary to cardiac microvascular damage^4^. ANS dysfunction with reduced heart rate variability (HRV) potentiates endothelial injury and accelerates the development of myocardial fibrosis in SSc patients, although the latter effect may appear early in the disease [6].

The 6-min walk test (6MWT) is a simple, inexpensive, easy-to-administer, well-tolerated, safe, noninvasive, and reliable submaximal test [7]. The 6MWT has been increasingly used to assess the exercise performance of SSc patients as a monitoring tool and as a primary measure of outcome and response to therapy [1]. The 6MWT is highly reproducible and can assess the overall prognosis of patients with SSc, in addition to better reflecting the ability to perform activities of daily living than other stress tests [8]. Even so, doubts remain about the relevance of the 6MWT for patients with SSc due to the involvement of SSc with multiple organs, including the heart [8].

ANS dysfunction at rest seems to be associated with worse exercise tolerance, even in patients with SSc without apparent cardiac involvement [4], although ANS dysfunction during exercise in these patients has not yet been studied. As low HRV is related to an increased risk of arrhythmic complications, the evaluation of HRV in patients with SSc during the 6MWT may help in the early detection of cardiac problems. Considering that sympathovagal imbalance is a potent risk factor for dangerous cardiovascular events and mortality, we believe that the possible interrelationship between HRV parameters and exertion may be a useful indicator in the evaluation of prognosis and risk stratification of individuals with SSc. Thus, the objective of this study was to evaluate the associations between sympathovagal balance and 6MWT performance in women with SSc without cardiac involvement.

## Methods and materials

Between May 2020 and February 2023, 69 women with SSc (out of 83 eligible) were recruited for a cross-sectional study. Those aged ≥ 18 years were recruited at the Hospital Universitário Pedro Ernesto of the Universidade do Estado do Rio de Janeiro, Rio de Janeiro, Brazil. A rheumatologist performed a thorough chart review to evaluate the accuracy of the SSc diagnosis. The patient was considered to have SSc if they fulfilled the 2013 revised American College of Rheumatology/European League Against Rheumatism (ACR/EULAR) classification criteria for SSc [9] with a score of 9 or higher. The following exclusion criteria were used: heart failure with reduced and/or preserved ejection fraction; valvular heart diseases; cardiac arrhythmias and conduction disorders; use of beta-blockers; inability to walk; and inability to perform the 6MWT. Subjects with relevant systemic comorbidities, such as a history of uncontrolled hypertension, diabetes mellitus, dyslipidemia, cerebrovascular and peripheral vascular diseases, hepatic or thyroid dysfunction, anemia, coagulopathy, and pregnancy or breastfeeding, were also not eligible. All patients underwent a basic clinical examination and routine laboratory testing. Cardiac involvement was excluded based on the absence of physical findings suggestive of cardiac disease, a normal electrocardiogram, and a normal transthoracic echocardiogram.

All participants signed a consent form, and the protocol was approved by the Research Ethics Committee of the Hospital Universitário Pedro Ernesto of the Universidade do Estado do Rio de Janeiro, Rio de Janeiro, Brazil, under number CAAE-52759521.2.0000.5259.

### Data collection procedure

The 6MWT was performed as previously described [10]. Briefly, the participants were instructed to walk the greatest possible distance in 6 min on a flat 30-m stretch, which was marked on the ground with cones at both ends. The 6MWT was preceded and followed by measurement of blood pressure (BP), heart rate (HR), respiratory rate, and peripheral oxygen saturation (SpO_2_). The 6MWT was immediately discontinued if SpO_2_ was < 80% or if exhaustion, chest pain, intolerable leg cramps, or diaphoresis was observed. The 6MWT tests were performed in duplicate with a 30-min interval between them, and each participant’s longest 6-min walking distance (6MWD) was compared to the predicted value [11].

Throughout the 6MWT, HRV was assessed using specific software (V800, Polar OY, Finland). The signs of normal-to-normal RR (NN) intervals captured by the cardiac monitor were exported to Kubios HRV software (Kuopio, Finland) for HRV analysis through time/frequency domain measurements and Poincaré plot nonlinear analysis. Measures in the time-domain analysis were as follows: mean NN interval; maximum HR; standard deviation of all NN intervals (SDNN), which captures the total HRV and reflects the circadian heart rhythm; root mean square of the difference between the coupling intervals of adjacent NN intervals (rMSSD), which correlates with the activity of the parasympathetic nervous system (PNS); number of successive NN interval differences > 50 ms (NN50), which primarily represents vagal activity; and the triangular interpolation of NN intervals histogram (TINN), which represents the global autonomic activity. Total power (0.04–0.15 Hz), which reflects global autonomic activity, was measured in the frequency domain analysis, and it was subdivided into the low-frequency range [LF, (0.04–0.15 Hz)], which is predominantly a marker of sympathetic nervous system (SNS) activity, and the high-frequency range [HF, (0.15–0.40 Hz)], which reflects the modulation of PNS efferent activity by ventilation. The LF/HF ratio, which reflects the autonomic balance, was also measured in the frequency domain analysis; a higher LF/HF ratio indicates a predominance of the SNS. LF and HF power were evaluated in normalized units (nu). Finally, the following nonlinear measures were evaluated with a Poincaré plot: standard deviation, measuring the dispersion of points in the plot perpendicular to the line of identity (SD1), which describes the short-term variability (represents parasympathetic modulation); standard deviation, measuring the dispersion of points along the line of identity (SD2), which describes the long-term variability (represents global cardiac autonomic activity); the SD2/SD1 ratio; and approximate entropy, which detects changes over time, indicating the complexity of the ANS. Registration and analysis were performed as recommended by the Task Force of the European Society of Cardiology and the North American Society of Pacing and Electrophysiology [12].

### Data analysis procedure

Data were analyzed with SPSS software, version 26. The Shapiro‒Wilk test was applied to verify the hypothesis of normality of the variables. The associations between the clinical variables of the 6MWT and HRV were analyzed using Spearman’s correlation coefficients. The results are expressed as median (interquartile range) or frequency (percentage). Differences were considered significant when *P* < 0.05.

## Results

Among the 83 women who were evaluated for inclusion in the study, five were excluded due to heart disease, four due to uncontrolled hypertension, three due to diabetes mellitus, and two due to inability to perform the 6MWT. The median age and time since diagnosis were 50 (39–59) and 9.5 (4–15) years, respectively. Participants’ lung function parameters were below the predicted values for normal lung function [13, 14]. None of the participants reported regular physical activity before the evaluation. Demographic, clinical, serological and pulmonary function data of the sample are shown in Table 1.

**Table 1:** Demographic and clinical data of the evaluated sample (*n* = 69)

Variables	Values
**Demographic data**	
Age (years)	51 (40–63)
BMI (kg/m^2^)	26.4 (24–31)
**Clinical characteristics**	
ILD (n, %)	30 (43.5%)
Gastrointestinal symptoms (n, %)	26 (37.7%)
Renal crisis (n, %)	6 (8.7%)
**Serology**	
Anti-TOPO I positivity (n, %)	43 (62.3%)
Anti-RNAP III positivity (n, %)	22 (31.9%)
Anti-centromere positivity (n, %)	19 (27.53%)
**Lung function**	
FVC (% predicted)	73 (62–86)
DLCO (% predicted)	58 (50–71)

*BMI* body mass index, *ILD* interstitial lung disease (diagnoses by computed tomography), *Anti-TOPO I* antibodies against topoisomerase I, *Anti-RNAP III* antibodies against RNA polymerase III, *FVC* forced vital capacity, *DLCO* diffusing capacity for carbon monoxide;

Results expressed as the median (interquartile range) or number (%).

The median 6MWD was 451 (392–498) meters, and 29 (42%) participants did not achieve 80% of the predicted value. Desaturation during the 6MWT (SpO_2_ ≤ 4%) was observed in 7 (10.1%) participants. The 6MWT data and the HRV measurements obtained during the 6MWT are shown in Table 2.

**Table 2:** 6-min walk test and heart rate variability data of patients with systemic sclerosis (*n* = 69)

Variables	Values
**6MWT**	
6MWD (m)	451 (392–498)
**Heart rate variability**	
Maximum HR, bpm	130 (117–144)
Mean NN intervals, ms	506 (471–572)
SDNN, ms	8.5 (5.8–16)
rMSSD, ms	7.2 (4.9–20)
NN50, ms	2 (0–9.5)
TINN, ms	77 (51–186)
TP, ms^2^	26 (9.5–90)
LF, ms^2^	14 (6–72)
LF, nu	75.3 (43–87)
HF, ms^2^	4 (1–32)
HF, nu	23.8 (13–56)
LF/HF	4.3 (0.9–9.1)
SD1, ms	5.1 (3.4–14.2)
SD2, ms	10.9 (7.1–18.3)
SD2/SD1	2 (1.2–2.7)
ApEn	1.2 (1–1.3)
PNS index	-2.8 (-3.3–-2.1)
SNS index	6.5 (4.5–9.2)

*6MWT* 6-min walk test, *6MWD* 6-min walking distance, *HR* heart rate, *NN* normal-to-normal RR intervals, *SDNN* standard deviation of all NN intervals, *rMSSD* root mean square of the difference between the coupling intervals of adjacent NN intervals, *NN50* number of interval differences of successive NN intervals greater than 50 ms, *TINN* triangular interpolation of NN intervals histogram, *TP* total power, *LF* low-frequency range, *HF* high-frequency range, *SD1* measuring the dispersion of points in the plot perpendicular to the line-of-identity, *SD2* standard deviation, measuring the dispersion of points along the line-of-identity, *ApEn* approximate entropy; *PNS* parasympathetic nervous system, *SNS* sympathetic nervous system.

Results expressed as the median (interquartile range) or number (%).

**Fig. 1:**
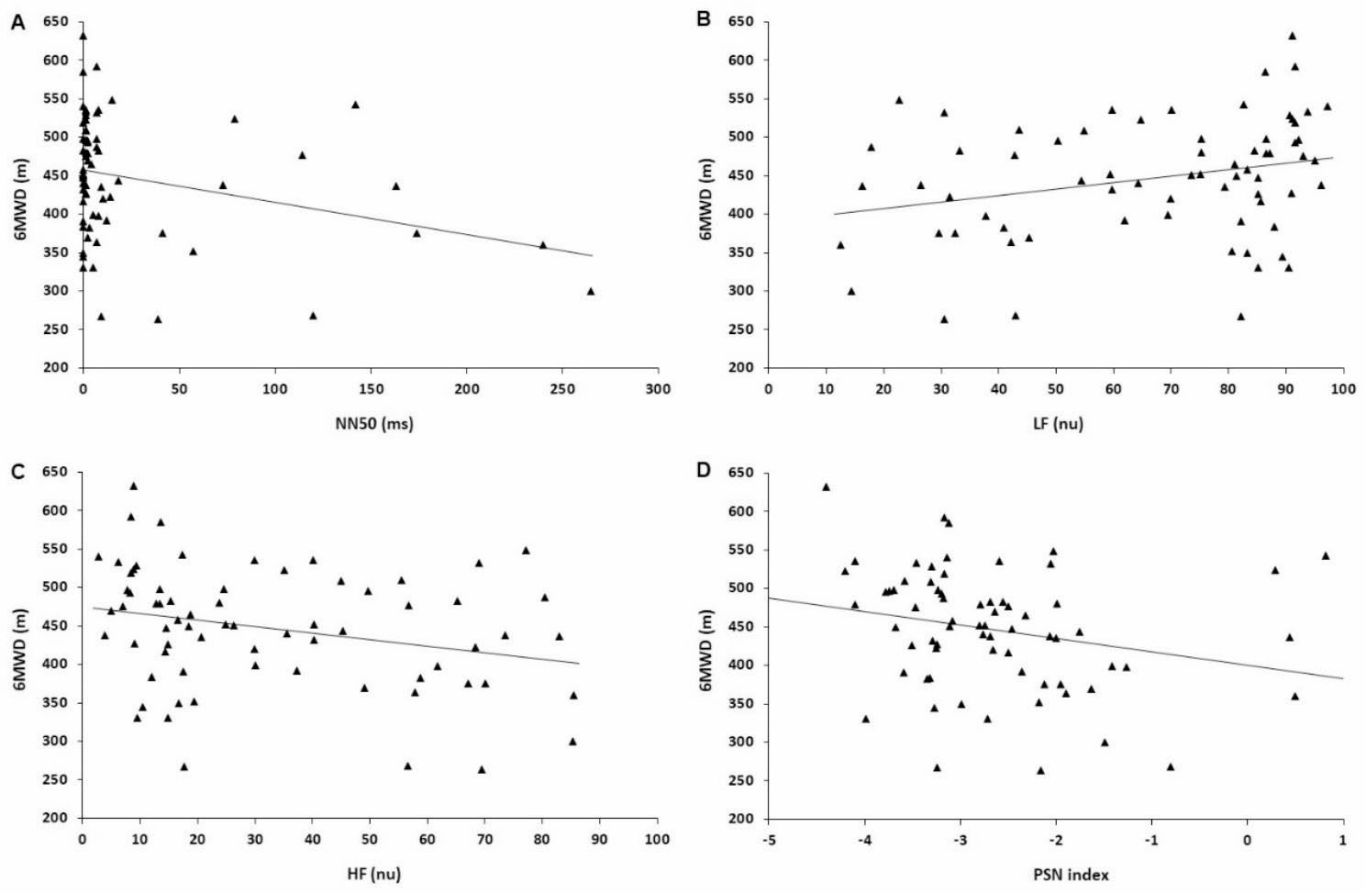
Relationships of 6-minute walking distance (6MWD) with the number of interval differences of successive normal-to-normal RR intervals greater than 50 ms (NN50, *r*_*s*_=-0.397, *P* = 0.013) **(A)**, low frequency range (LF, *r*_*s*_=0.374, *P* = 0.023) **(B)**, high frequency range (HF, *r*_*s*_=-0.372, *P* = 0.023) **(C)**, and index parasympathetic nervous system (PNS, *r*_*s*_=-0.342, *P* = 0.045) **(D)** in patients with systemic sclerosis

The interrelationships between the 6MWT and HRV parameters are shown in Fig. 1. We observed significant correlations between the 6MWD and the following HRV parameters: NN50 (*r*_*s*_=-0.397, *P* = 0.013), LF (*r*_*s*_=0.374, *P* = 0.023), HF (*r*_*s*_=-0.372, *P* = 0.023), and PNS index (*r*_*s*_=-0.342, *P* = 0.045). No significant correlation was found between delta SpO_2_ (pre- and post-6MWT) and HRV parameters.

## Discussion

People with SSc may present with subclinical involvement of the cardiovascular system, which can have a significant impact on functional capacity [15]. In this sense, HRV analysis is a powerful noninvasive tool for assessing the sympathetic and vagal modulations of the heart, in addition to being simple to apply and widely available [5]. Evaluating ANS behavior during the 6MWT, we observed a relationship between both PNS withdrawal and SNS activation and the 6MWT measures. To our knowledge, this is the first study to evaluate HRV during a submaximal test in patients with SSc.

Patients with SSc have difficulty performing activities that require physical effort. In this regard, it is important to note that vasculopathy causes inadequate blood flow to the cardiopulmonary system, which, together with musculoskeletal limitations, results in exercise intolerance and other exercise limitations [16]. Transthoracic echocardiography was used to identify associations between a lower 6MWD and a reduced left ventricular ejection fraction or pulmonary arterial systolic hypertension in patients with SSc [8, 17, 18]. Since we excluded patients with abnormal transthoracic echocardiograms, it is possible that obvious cardiac structural changes do not explain our results.

Although the overall long-term prognosis of patients with SSc has improved in recent years, the proportion of deaths from heart disease has not changed significantly^8^, so monitoring cardiac involvement is essential in the management of these patients. We observed a direct relationship between LF—which is a marker of sympathetic hypertonia—and 6MWD. In line with our results, Di Paolo et al. [4] observed positive correlations of LF and LF/HF with peak oxygen uptake (VO_2peak_) in SSc patients without pulmonary hypertension who underwent cardiopulmonary exercise testing and monitoring with a 24-hour Holter monitor. Those authors showed that the LF/HF ratio was the only independent predictor of VO_2peak_, explaining 25% of its variability. Using 24-h Holter monitoring in women with SSc, Poliwczak et al. [6] observed that women with SSc had lower LF than healthy controls, suggesting that sympathetic hyperactivation may disturb the balance between vasoconstriction and vasodilation in favor of the former. Since sympathetic hypertonia is associated with increased cardiovascular workload, endothelial dysfunction and coronary spasm, it is clinically important to further investigate these patients for possible subclinical cardiovascular damage [5].

An increase in vagal activity exerts a protective effect against ischemia and decreases HR and BP [5]. In the present study, however, we observed a negative correlation between the 6MWD and several parameters of vagal modulation, including the NN50, HF, and PNS index. The PNS index is computed in Kubios HRV software using the mean NN interval, rMSSD, and Poincaré plot index SD1 [12]. Altogether, our findings reinforce the hypothesis that in patients with SSc, ANS dysfunction is related to an impairment in vagal regulatory outputs, with consequent reduction of baroreflex activity and SNS activation. Although the pathophysiological mechanisms are not yet fully understood, the SNS hyperactivity observed in these individuals can be explained at least in part as a compensatory response to the cardiac microvascular changes present even in the early stages of the disease. Notably, if it is not modulated, microvascular damage per se may be responsible for the progression of SSc-associated vasculopathy and the chronic complications that occur in SSc [4]. The link between the ANS and persistent low-grade chronic inflammation appears to be bidirectional in SSc, and SSc may result in the deterioration of the microcirculatory system and thereby compromise cardiac structures [5].

### Limitations

The study has limitations. First, it was performed in a relatively small population, although the sample was homogeneous, and this is a rare condition. Second, a control group would have allowed direct comparisons between the measured variables, although the discrepancies are enormous between SSc patients and healthy controls. Indeed, the absence of a control group was minimized by the severity of SSc, which was highlighted by the patients’ low test results compared to the predicted values for the healthy population. In this sense, our patients had low lung-function values compared with the predicted values for healthy individuals (which is a characteristic of SSc) [19], and almost half of them had a 6MWD that was less than 80% of the predicted value when normalized to anthropometric data, such as age, height and body mass. Third, we used data only from females, although this is in agreement with the general epidemiological findings of SSc, which indicate a clear predominance in women [20]. Finally, our assessment of SpO_2_ using fingertip pulse oximetry may have impacted the correlational analysis, as the preferred method for monitoring SpO_2_ in patients with SSc involves the use of forehead pulse oximetry because the presence of Raynaud’s phenomenon makes finger and earlobe pulse oximetry readings unreliable [15]. Despite these limitations, cardiac autonomic balance may become a target in the treatment of SSc patients. Thus, further studies with larger cohorts using comparisons with healthy controls and longitudinal assessments are needed to validate our findings and investigate whether the associations between sympathovagal balance and exercise performance provide clinically important contributions in the long term.

## Conclusion

In conclusion, in women with SSc, there is an interrelationship of 6MWD with both vagal withdrawal and sympathetic hyperactivation. This relationship between autonomic imbalance and reduced exercise performance can increase cardiovascular risk, even in patients who were assessed by transthoracic echocardiography and found to be without cardiac involvement. Thus, drug- and nondrug treatments that reduce sympathetic hypertonia and prevent parasympathetic withdrawal should be considered to counteract autonomic dysfunction in SSc.

## Data Availability

All the data supporting the results are provided in the manuscript.

## Abbreviations

6MWD: 6-minute walking distance
6MWT: 6‐minute walk test
ANS: autonomic nervous system
ApEn: approximate entropy
BP: blood pressure
HF: high-frequency range
HR: heart rate
HRV: heart rate variability
LF: low-frequency range
NN: normal-to-normal RR intervals
NN50: number of successive NN interval differences > 50 ms
PNS: parasympathetic nervous system
rMSSD: root mean square of the difference between the coupling intervals of adjacent NN intervals
SD1: standard deviation, measuring the dispersion of points in the plot perpendicular to the line-of-identity
SD2: standard deviation, measuring the dispersion of points along the line-of-identity
SDNN: standard deviation of all NN intervals
SNS: sympathetic nervous system
SpO_2_: peripheral oxygen saturation
SSc: systemic sclerosis
TINN: triangular interpolation of NN intervals histogram
TP: total power
VO_2peak_: peak oxygen uptake
